# Uncommon cause of fungemia in a patient with renal cell cancer: A case report of *Candida lusitaniae* Fungemia: Erratum

**DOI:** 10.1097/MD.0000000000010275

**Published:** 2018-03-23

In the article, “Uncommon cause of fungemia in a patient with renal cell cancer: A case report of *Candida lusitaniae* Fungemia”,^[1]^ which appeared in Volume 96, Issue 45 of *Medicine*, Dr. Puvanalingam Ayyadurai's first name appeared incorrectly as Puvvalingam.
